# Practical Notes and Formulæ

**Published:** 1885-05-20

**Authors:** 


### PRACTICAL NOTES AND FORMULÆ.

**An Inhalation for Coryza.**—Hager (Union med.) recommends the following formula:

℞ Pure carbolic acid.............................grs. cl

Ammonia water.............................ʒ iij

Alcohol.............................℥ j

Distilled water.............................ʒ v.

A piece of cotton saturated with this mixture is placed in a wide-mouthed bottle, and the vapor is inhaled.—*New York Med. Jour.*

**For Ring-Worm.**—Hyposulphite of soda lotion (ʒ j or ʒ jss to the ounce) is one of the most successful applications to ring-worm, though in some cases oleate of mercury acts more expeditiously. Oleate of copper is objectionable on account of its brilliant color; moreover, it did not answer expectations under protracted trial at Middlesex Hospital, London. Hairdressers unwittingly propagate ringworm to a considerable extent by using the same brush and comb for different children. The hyposulphite lotion, above-mentioned, is especially effective in chloasma.—*Med. World.*

**For Gonorrhœa.**—Dr. F. E. Michael (Baltimore) highly recommends the following mixture in gonorrhœa and its vesical complications:

℞ Ol. santal,

Liq. potassæ,

Ætheris nit.,

Tinct. cinnamoni, } aa.............................℥ ss

Mucilag. sem. lini.............................℥ iv.

M. Sig. One dessertspoonful three times a day.—*Med. World.*

**Paper for Taking Out Ink Stains.**—This is a thick blotting paper, soaked in a saturated solution of oxalic acid and dried. When this is laid upon a fresh blot, it removes it and does not leave a stain. The poisonous character of this paper should not be forgotten.—*National Druggist.*

**Ginger Ale Easily Made.**—

℞ Brown sugar.............................℔ ij
Boiling water.............................cong. ij
Cream of tartar.............................℥ j
Bruised ginger root.............................℥ ij.

Infuse the ginger in boiling water; add your sugar and cream of tartar; when lukewarm, strain; then add ½ pint good yeast; let it stand all night, then bottle. If you desire, you can add 1 lemon and the white of an egg to fine it.—*Scientific American.*

**White's Cough Syrup.—**

℞. Syr. tolu.................................... 2 ozs.
Syr. squills comp.............................. 6 ozs.
Syr. ipecac.................................... 1 oz.
Glycerin....................................... 4 ozs.
Tint. lobelia.................................. 6 ozs.
Tinct. opium camph............................. 6 ozs.
Extr. pilocarpus fl............................ 2 ozs.
Ammonium chlorid............................... 1 oz.

M. Take a teaspoonful three times during the day, and every hour or two before going to bed.—*Medical Age.*

**Lusk's Aperient Pills:**

℞. Ext. aloes socot............................ ℈i.

Pulv. rhæi..................................... gr. x.

Ext. nuc. vom.................................. gr. x.

Ext. tarax..................................... ʒss.

M. Fiant pil. No. XX. S. One before meals.— *Western Med. Reporter.*

**Lotion for Dandruff:**

℞. Tinct. capsic............................... 2 p.
Glyc........................................... 8 p.
Cologne........................................ 2 p.
Aq............................................. 25 p.

M. S. Apply, by means of a sponge, to the scalp, every day. — *Western Medical Reporter.*

**Dr. Sabonblene's Benzoated Ptisan.**—In renal and vesical affections when the urine is turbid or muddy, Dr. S. uses with the greatest benefit:

℞. Benzoic acid................................ 15 to 30 grs.
Aromatic distilled water....................... 2 ozs.
Sugar.......................................... 4 ozs.
Water q. s. ad................................. 32 ozs.

M. Drink freely.—*Medical Age.*

**Coryza.**—Robinson ( *Western Medical Reporter*) recommends the following:

℞. Pulv. belladon.............................. gr. xx
Pulv. morph. sulph............................. gr. ii.
Gum acac. *ad*................................. ʒss.

M. This is used freely as a snuff during the day.

**Remedy Against Distressing and Fatiguing Coughs.**—Take one ounce and a half or two ounces of pure glycerine and evaporate in a porcelain capsule, by means of a spirit lamp. An enormous amount of vapor is disengaged, which inhaled, brings relief to persistent coughs.—*Revue Medicale.*

